# Fungal Ribotoxins: A Review of Potential Biotechnological Applications

**DOI:** 10.3390/toxins9020071

**Published:** 2017-02-21

**Authors:** Miriam Olombrada, Rodrigo Lázaro-Gorines, Juan C. López-Rodríguez, Álvaro Martínez-del-Pozo, Mercedes Oñaderra, Moisés Maestro-López, Javier Lacadena, José G. Gavilanes, Lucía García-Ortega

**Affiliations:** 1 Departamento de Bioquímica y Biología Molecular I, Facultad de Química, Universidad Complutense de Madrid, 28040 Madrid, Spain; miriam.olombrada@ieu.uzh.ch (M.O.); rodrigolazarogorines@ucm.es (R.L.-G.); jc.lopez@ucm.es (J.C.L.-R.); alvaromp@quim.ucm.es (A.M.-d.-P.); monaderr@bio.ucm.es (M.O.); moisesmaes@gmail.com (M.M.-L.); jlacadena@quim.ucm.es (J.L.); jggavila@ucm.es (J.G.G.); 2 Department of Evolutionary Biology and Environmental Studies, University of Zurich, Winterthurerstrasse 190, 8057 Zurich, Switzerland; 3 Departamento de Estructura de Macromoléculas, Centro Nacional de Biotecnología (CNB-CSIC), Darwin 3, 28049 Madrid, Spain

**Keywords:** fungal ribotoxins, ribonuclease (RNase), ribosome, sarcin-ricin loop (SRL), insecticide, biopesticide, immunotoxin, cancer, ribosomopathies

## Abstract

Fungi establish a complex network of biological interactions with other organisms in nature. In many cases, these involve the production of toxins for survival or colonization purposes. Among these toxins, ribotoxins stand out as promising candidates for their use in biotechnological applications. They constitute a group of highly specific extracellular ribonucleases that target a universally conserved sequence of RNA in the ribosome, the sarcin-ricin loop. The detailed molecular study of this family of toxic proteins over the past decades has highlighted their potential in applied research. Remarkable examples would be the recent studies in the field of cancer research with promising results involving ribotoxin-based immunotoxins. On the other hand, some ribotoxin-producer fungi have already been studied in the control of insect pests. The recent role of ribotoxins as insecticides could allow their employment in formulas and even as baculovirus-based biopesticides. Moreover, considering the important role of their target in the ribosome, they can be used as tools to study how ribosome biogenesis is regulated and, eventually, may contribute to a better understanding of some ribosomopathies.

## 1. Introduction

Organisms populate almost any ecological niche of the planet and establish a high variety of biological interactions, ranging from mutualism to antagonism, mainly in search for resources to survive (light, nutrients, water, etc.). In particular, fungi constitute a rich source of nitrogen and phosphorous for arthropods and are under constant attack by fungivores such as collembolan, mites and insects [1]. However, they can also present mutualistic relationships like those established with fungus farming ants. Thus, a complex network of interactions is established in nature involving predatory, defensive and mutualistic relationships. In this context, fungi produce a great variety of toxins, many of them are proteins. These toxins are secreted with many different purposes, although self-defense and fungi development have been suggested to be their principal functions.

**Figure 1:**
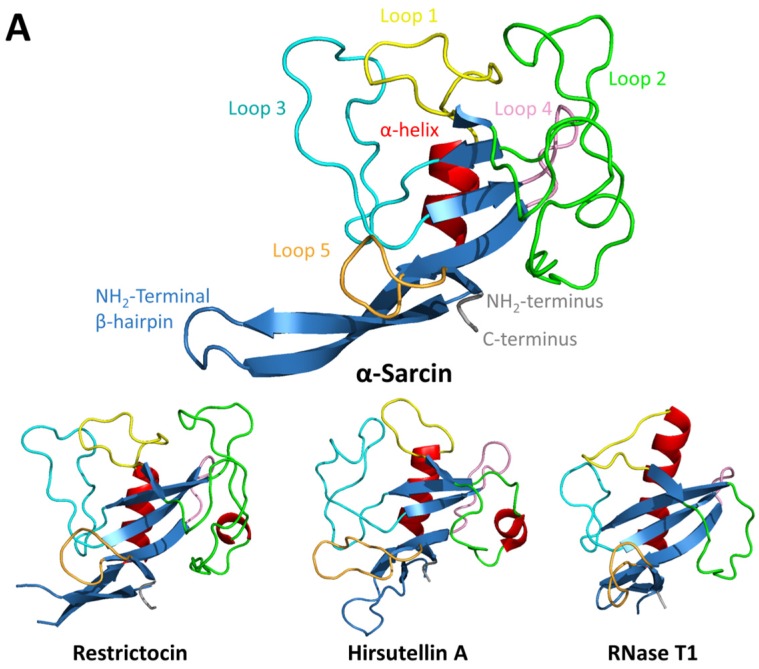

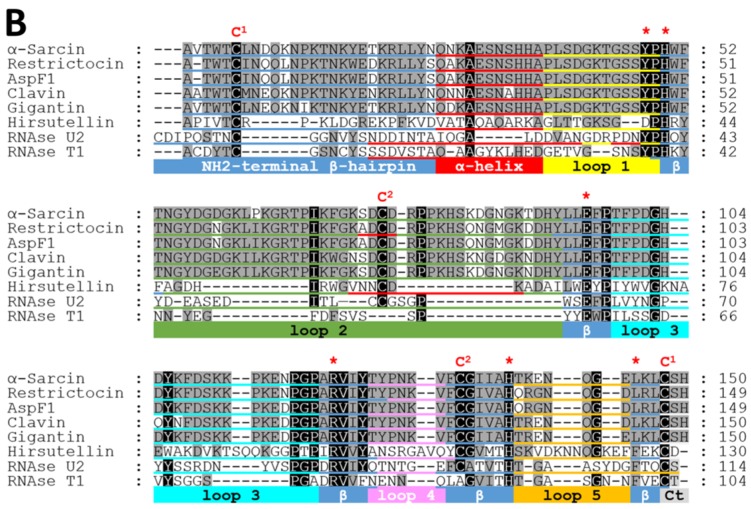
Ribotoxins structure and sequence features. (**A**) The three dimensional structures of the three most representative ribotoxins and RNase T1 are shown (PDB IDs: 1DE3, 1AQZ, 2KAA, 9RNT). In α-sarcin elements of secondary structure are shown. Diagrams were generated with PyMOL [20]; (**B**) Sequence alignment of the most representative fungal RNases from the RNase T1 family. Conserved Cys forming disulphide bridges [C] as well as the active site residues [*] are indicated. Conserved amino acids (light grey boxes) in at least seven sequences are highlighted in black. Elements of secondary structure are displayed by the same colors as in A.

**Figure 2:**
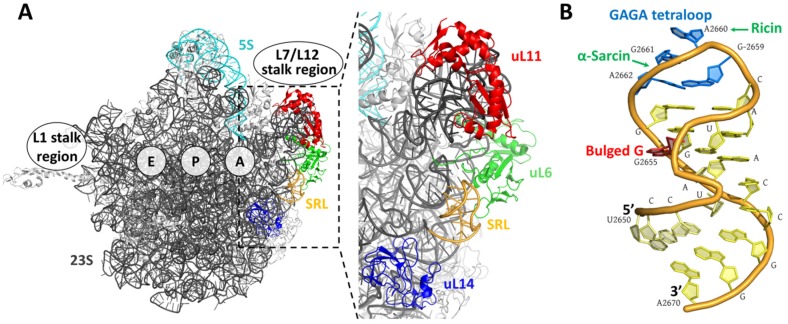
The target of ribotoxins: the ribosome. (**A**) Three-dimensional structure of the large ribosomal subunit of *Escherichia coli* (PDB ID: 2AW4). The location of L1 and L7/L12 stalks (absent in this crystal) and E, P and A sites are indicated. Conserved proteins around the SRL (orange) appear in different colors: uL6 (green), uL11 (red), uL14 (blue). Other ribosomal proteins appear in light gray. 23S (dark gray) and 5S (cyan) rRNAs are also shown; (**B**) SRL structure. The bulged G (red), the GAGA tetraloop (blue), the bond cleaved by α-sarcin and the adenine depurinated by ricin, are indicated.

Fungal ribotoxins are a family of extracellular ribonucleases that have been studied in detail since their discovery in the early 1960s. They are not the only extracellular ribonucleases (RNases) produced by filamentous fungi, but are the only ones shown to display cytotoxic activity (Figure 1). Ribotoxins are produced by several fungi species, mostly from the genus *Aspergillus*, although other entomopathogenic fungi such as *Hirsutella thompsonii* or *Metarhizium anisopliae* also produce them [2,3,4,5,6]. They are highly specific, exerting their ribonucleolytic activity on the larger RNA molecule (rRNA) of the ribosome. From this perspective, they could be included in the group of ribosome-inactivating proteins (RIPs), however, this term usually refers to those toxins with N-glycosidase activity and plant origin, best represented by ricin, a toxin found in the seeds of the castor bean plant *Ricinus communis*. Both RIPs and ribotoxins target the same universally conserved sequence of rRNA, the so-called sarcin-ricin loop (SRL), by either depurinating a single nucleotide or cleaving a unique phosphodiester bond, respectively (Figure 2). However, the mechanisms employed by both types of toxins to reach their target are quite different [7,8].

Even though the biological function of ribotoxins has not been cleared up yet, several studies have shown the insecticidal properties of at least two of these proteins, supporting their involvement in defense and parasitism [9,10,11]. The detailed study of the structure and function of this family of toxic proteins has highlighted their potential use in biotechnological applications, either as bioinsecticides to control insect pests [12] or as antitumor agents in human therapies [13,14]. Moreover, they could also be used as specific tools for the study of human ribosome-related diseases, the so-called ribosomopathies [15,16].

## 2. Fungal Ribotoxins

Ribotoxins are not the only RNases produced by fungi. Indeed, they are part of a much wider group of fungal extracellular RNases. Ribotoxins are larger than non-toxic fungal RNases but still small proteins of about 150 amino acids, and generally basic. The main structural difference between non-toxic RNases, such as RNases T1 or U2, and ribotoxins would be that the latter contain longer and positively charged loops, which have been suggested to be the structural basis of their toxicity [17] (Figure 1). Identification of these structural features that make ribotoxins such efficient natural killers would be a major step towards their utilization as specifically targeted weapons against insect pests or different human pathologies. So far, the best characterized ribotoxins are α-sarcin, restrictocin, Aspf1, and hirsutellin A (HtA). The first three, produced by different *Aspergillus* spp., show a high degree of conservation with sequence identities above 85%. However, HtA, a ribotoxin produced by the entomopathogenic fungus *Hirsutella thompsonii*, only shares 25% of sequence identity with them (Figure 1B). This observation confirmed that ribotoxins spread across fungal species, and are not only restricted to the genus *Aspergillus* [2,3,18,19].

The ribonucleolytic activity of ribotoxins is extremely specific, targeting a sequence of rRNA, the SRL, highly conserved in all ribosomes (Figure 2). This sequence is located on the larger molecule of rRNA of the large ribosomal subunit. During translation, the SRL together with the uL11-binding region, the L7/L12 stalk (P1/P2 in yeast) and ribosomal proteins uL6 and uL14, constitute the elongation factor-binding site that is required for correct functioning of the ribosome [21,22]. The SRL structure consists of a distorted hairpin, and the most significant structural features are a GAGA tetraloop and a bulged G motif. Ribotoxins cleave a single phosphodiester bond of the GAGA tetraloop, producing a small fragment of 300–400 nucleotides of rRNA (depending on the ribosome origin), known as the α-fragment (Figure 2). They are cyclizing RNases displaying the general acid-base type endonucleolytic cleavage of RNA mechanism in two steps, just like other members of the RNase T1 family [4,23]. Cleavage of this phosphodiester bond inhibits protein biosynthesis and produces cell death by apoptosis [24].

The three-dimensional structure of several ribotoxins is known at atomic resolution (Figure 1A) [25,26,27], and mutational analyses have allowed the assignment of specific roles to different residues (implication in the catalysis, ribosome and SRL recognition, interaction with the cell membrane, etc.). In the case of α-sarcin, the catalytic residues are His 50, Glu 96 and His 137 (Figure 1B). These three residues are required for the specific inactivation of ribosomes. Additional mutational studies showed that Tyr 48, Arg 121 and Leu 145, although not essential, would also contribute to the ribotoxin activity of α-sarcin [28,29,30] (Figure 1B). The equivalent residues in HtA (His 42, Glu 66 and His 113) were identified by structural comparison, showing that the catalytic residues are conserved, but also presenting other features closer to the T1-like RNases (Phe 126 instead of the equivalent Leu 145, for example) or even some others which were completely new (Asp 40 instead of Tyr 48) [31] (Figure 1B). Mutagenesis studies have indeed revealed that the active site of HtA would provide a more adaptable microenvironment than the one described for α-sarcin being able to better accommodate electrostatic and structural changes [31,32,33].

The toxicity of ribotoxins results from the combination of their highly specific RNase activity and their ability to cross membranes. So far, no protein receptor for ribotoxins has been found in the cell. For that reason, even though ribotoxins can potentially inactivate any ribosome, the lipid composition of cell membrane plays an important role in their cytotoxic specific activity. Accordingly, it has been described how ribotoxins are more efficient on transformed or virus-infected cells, most likely due to altered permeability and/or composition of their cell membrane [24,34]. In insects, lipidic membrane is thinner and more fluidic than in other eukaryotes, therefore, more accessible and sensitive to ribotoxins [11,35]. The use of lipid model systems has proven that α-sarcin interacts with lipid vesicles enriched in acidic phospholipids. This interaction causes vesicle aggregation followed by fusion and intermixing of phospholipids and, finally, leakage of their aqueous contents [36,37]. However, this mechanism does not seem to be conserved among ribotoxins. HtA does not promote vesicle aggregation even though it shows a higher membrane-permeabilizing ability than α-sarcin in leakage experiments [5,11]. In α-sarcin, the region comprising residues 116–139 was thought to participate in the hydrophobic interaction with membranes [38,39] and recently it was proposed that Lys residues 111 and 114, in loop 3, would also take part in the electrostatic interactions needed to bring vesicles into contact [40]. For HtA, a role in membrane-permeabilizing activity has been proposed for Trp71 and Trp78 [32]. The ability to interact with lipid membranes has also been associated with the N-terminal β-hairpin of ribotoxins. Deletion of this positively charged region in α-sarcin produces a non-toxic but active ribonuclease with altered membrane interaction properties [41]. It is in this region where HtA and restrictocin show more variability when compared to α-sarcin. The N-terminal β-hairpin of HtA is much shorter (Figure 1), but this truncation appears to be compensated by the extension of loop 5, which also exhibits a higher amount of positive charges [32,33].

The positively charged surface of ribotoxins allows them to establish favorable electrostatic interactions with the ribosome [42,43,44]. Moreover, the high specificity of ribotoxins requires of additional elements besides the active site capable of interacting with the ribosome to recognize the SRL. So far, several regions have been proposed to participate in the specific recognition of the SRL and other ribosomal elements. The Lys-rich region of loop 3 may interact with a phosphodiester bond around the bulged G of the SRL, whereas residues 51–55 of loop 2 and some residues of loop 5 may contact the GAGA tetraloop that is cleaved by the toxin [42,45] (Figure 1 and Figure 2). Docking models using the *Haloarcula marismortui* ribosome and α-sarcin structures suggested that apart from interactions with the SRL, α-sarcin could interact with neighboring ribosomal proteins. A short sequence of loop 2 would contact ribosomal protein uL6 and the N-terminal β-hairpin, specifically the stretch 11–16, would interact with ribosomal protein uL14 (Figure 1 and Figure 2). In addition, other ribosomal regions involved in the recruiting of elongation factors during translation could also establish interactions with toxins. That is the case of the ribosomal stalk proteins and RIPs like ricin, trichosantin and Shiga-like toxins [46]. The ribosomal stalk is a protruding structure of the ribosome that serves as an anchoring platform for elongation factors and by several RIPs to further recognize the SRL. Despite sharing the same target, not all RIPs behave identically, since no interaction has been found between pokeweed antiviral protein and the eukaryotic stalk proteins [47]. In the case of ribotoxins, the eukaryotic ribosomal stalk does not participate in α-sarcin inactivation of the ribosome [8]. This result suggests that ribotoxins use a different mechanism of ribosome recognition than that used by plant RIPs like ricin.

Finally, besides the toxicity exerted by ribotoxins due to their catalytically inactivation of the ribosome, there are some studies highlighting the ability of α-sarcin to inhibit protein biosynthesis and promote cytotoxicity independently of its ribonucleolytic activity [48,49]. These observations suggest an alternative mechanism for cell death that still needs to be explored.

## 3. The Genus *Aspergillus* and Other Ribotoxin-Producer Fungi

Fungal ribotoxins were first discovered in the 1960s during a screening program searching for antibiotics and antitumor agents. The mold *Aspergillus giganteus* was found to produce a protein that could inhibit sarcoma and carcinoma induced in mice [50] which was named α-sarcin. Soon after, two other antitumor proteins with similar activity, restrictocin and mitogillin, were discovered in *A. restrictus*. More recent studies proved that all species assigned to *Aspergillus* section *clavati* contain ribotoxin genes [18]. *Aspergilli* are a ubiquitous and complex group of filamentous fungi containing more than 185 species including human pathogens, like *A. fumigatus*, as well as others used in industry for the production of food and enzymes, like *A. oryzae* [51]. By 2008, the genome of seven species of *Aspergillus* had been sequenced. This was a promising step towards the understanding of fungi biology and evolution. Indeed, sequencing the genome of other fungi could also be used for the identification of new species that produce ribotoxins. Moreover, the comparison of these genomes with those from non-producer species could shed new light on the biological function of these proteins.

**Figure 3:**
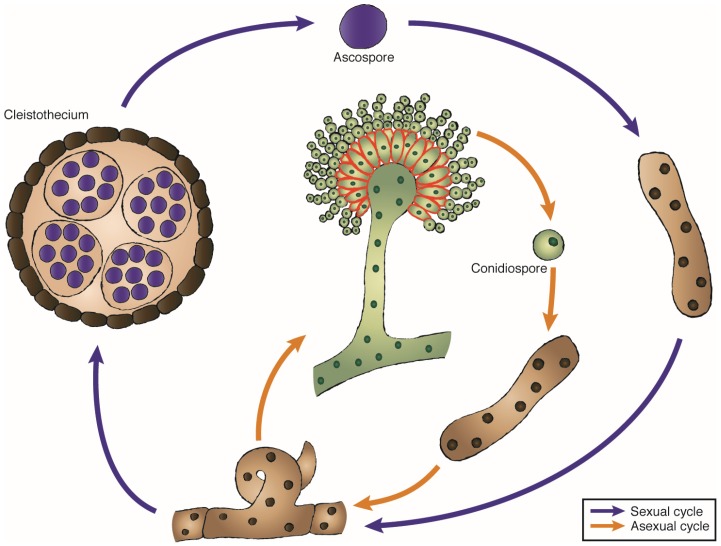
Life cycle of *Aspergillus* and suggested localization of ribotoxins. *Aspergillus* can enter a sexual or an asexual reproductive cycle. During the sexual cycle, the mycelium forms a fruiting body, the cleistothecium, which holds the ascospores that once released into the environment can form a new mycelium. In the asexual reproduction, the mycelium differentiates into identical asexual spores or conidiospores. Ribotoxins would be produced during maturation of the conidia, and would be located on the edge of the phialides (shown in red). Additionally, a parasexual cycle can take place in *Aspergillus* (not shown).

Most filamentous fungi have a complex life cycle. Like other Ascomycota, some *Aspergillus* spp. can have both sexual and asexual reproductive cycles (Figure 3). *A. nidulans* constitutes a good example to describe them since it can present both types of reproduction [52]. In the sexual reproduction, karyogamy (nuclei fusion) and meiosis take place. The fungus develops a fruiting body, the cleistothecium, which produces sexual spores or ascospores. During the asexual cycle, the mycelium, which develops from a single haploid spore, differentiates into many identical spores named conidia or conidiospores, which can form a new web of hyphae. There are only a few studies of ribotoxin expression by their natural host and its role in its life cycle. In those, the presence of ribotoxins has been detected during the asexual reproduction of *A. restrictus*. The ribotoxin restrictocin, which is almost indistinguishable from α-sarcin, is produced by *A. restrictus* at a very specific moment of fungal development, the initial steps of conidiophore formation, and it is degraded afterwards [53,54]. Furthermore, in solid medium restrictocin remained attached exclusively to the phialides and disappeared after conidia maturation (Figure 3). Plausible explanations for this pattern in the ribotoxin expression could be that restrictocin has a role in the process of conidiation, or a protective function of the maturing conidia from predators until the development is complete. Once conidiation is finished, restrictocin would be degraded so that fungus-feeding insects could externally or internally carry spores to new locations for spreading the fungus. Following the idea of a protective function of ribotoxins during maturation of conidia, there are some studies on the different feeding behavior of the fungivorous beetle *Carpophilus freemani* upon restrictocin production [9]. Feeding of *C. freemani* adults on *A. restrictus* decreased during conidia formation, something that was not observed when they fed on *A. nidulans* at the same developmental stage. This deterrence of insect feeding could be related to an increase in restrictocin production by *A. restrictus* and not by *A. nidulans*. Heterologous expression of restrictocin in *A. nidulans* also inhibited insect feeding by *C. freemani* beetles [10].

As mentioned before, the production of ribotoxins is not restricted to *Aspergillus* [2,3,6,18,19,55]. The genus *Hirsutella* includes over 50 fungal species that infect many species of invertebrates. The entomopathogenic fungus *H. thompsonii* also produces a ribotoxin. This fungus is known to cause natural epizootics in populations of eriophyid mites. Under in vivo conditions, the conidia of *H. thompsonii* attach to the cuticle of the host, germinate and penetrate through it. The hyphae then grow inside the insect and reproduce asexually, contacting new hosts to begin a new cycle of infection [56]. Studies of the toxicity and pathology of crude filtrates of this fungus during the 1990s revealed a high specificity for the subclass *Acari*, although it was also toxic for other arthropods like moth, flies and mosquito larvae [57]. The role of toxins in the pathogenesis of insect infection by *H. thompsonii* has been investigated as well. It was known that other entomopathogenic fungi like *M. anisopliae* and *Beauveria bassiana* produce cyclic peptides that are toxic to insect [58]. These peptides induced immediate paralysis and quick mortality when injected into *Galleria mellonella* larvae. Symptoms upon injection of *H. thompsonii* culture filtrates were different and rather slower, but still lethal for *G. mellonella* larvae. Several investigations led to the identification of hirsutellin A, a protein produced by *H. thompsonii var thompsonii* with insecticidal activity against *G. mellonella* and *Aedes aegypti* larvae [19,59]. Analysis of 162 strains of *H. thompsonii* revealed the presence of the HtA gene in 100 strains but the expression of HtA was poorly correlated with the mortality rates induced by broth filtrates in *G. mellonella*, suggesting that there could be additional toxic factors involved in the insecticidal activity of the fungus [60]. There are no studies on the exact localization of HtA in the fungus, like those performed with *A. restrictus.* Therefore the production of this toxin cannot be linked to a developmental stage yet. Soon after its discovery, HtA was related to ribotoxins due to its ability to specifically cleave rRNA on *Spodoptera frugiperda* cells (Sf9), inhibiting cell growth [19,55]. HtA was then subjected to a fine structural and functional characterization, demonstrating that despite its smaller size and low sequence identity, it was a ribotoxin [5,31,32,33,61].

In fact, characterization of HtA opened up the possibility of an insecticidal function of ribotoxins in general. This idea is also supported by the fact that *Aspergillus*, the main ribotoxin producer known so far, shares feeding niche with several insects and, as a way to compete for survival, it could have developed weapons like ribotoxins. Purified restrictocin included in the insects’ diet killed *C. freemani* and *S. frugiperda* larvae, and deterred feeding of adult *C. freemani* [9]. A detailed comparison of the activity of HtA and α-sarcin proved that the latter is nearly as toxic as HtA against insect larvae, insect cell cultures and insect ribosomes [11]. Biochemical characterization of the activity of ribotoxins against insect cells showed how HtA and α-sarcin cause protein synthesis inhibition and the release of the characteristic α-fragment resulting from the rRNA cleavage [11], just as it had been previously shown with ribosomes of different origin [4,5,21]. In the conditions assayed, HtA is significantly more active than α-sarcin against isolated insect ribosomes, suggesting that the passage across the cell membrane might be the rate-limiting step for their cytotoxic activity. Of course, in a natural environment additional factors may influence their efficiency, such as their natural biosynthesis and extracellular export, stability in a particular environment or accessibility to their target. Reinforcing this hypothesis about their insecticidal natural function, a new ribotoxin, very similar to HtA, has been recently cloned and characterized [6]. This small ribotoxin is produced by the fungus *M. anisopliae* and therefore has been named as anisoplin.

The fact that ribotoxins can inactivate every ribosome known raises the question of how fungi protect their own ribosomes [62]. Ribotoxins are synthesized as precursors that are processed inside cellular membrane compartments. Protection of the producing cells must rely on a very efficient recognition of the signal sequences and an adequate compartmentalization before they are secreted to the extracellular medium [4,63,64,65]. In fact, when the leader sequence was altered or changed by a different one, heterologous expression of restrictocin in *A. nidulans* showed reduced transcript levels and enhanced cellular lysis, and the ribotoxin was not always localized in the conidiophores of the fungus [66], suggesting that the native leader sequence of restrictocin is highly efficient in its role of protecting fungi during secretion. The restrictocin gene in *A. restrictus* has an intron at the beginning of its sequence [67]. Recently, a study has proven that introns can be used to attenuate toxicity of barnase for its use in suicide gene therapy [68], so perhaps the existence of an intron in restrictocin is an additional example of the strategies used by these fungi to attenuate ribotoxins’ toxicity against their own ribosomes during their synthesis and secretion to the extracellular medium.

## 4. The Potential Biotechnological Uses of Fungal Ribotoxins

### 4.1. Immunotoxins

**Figure 4:**
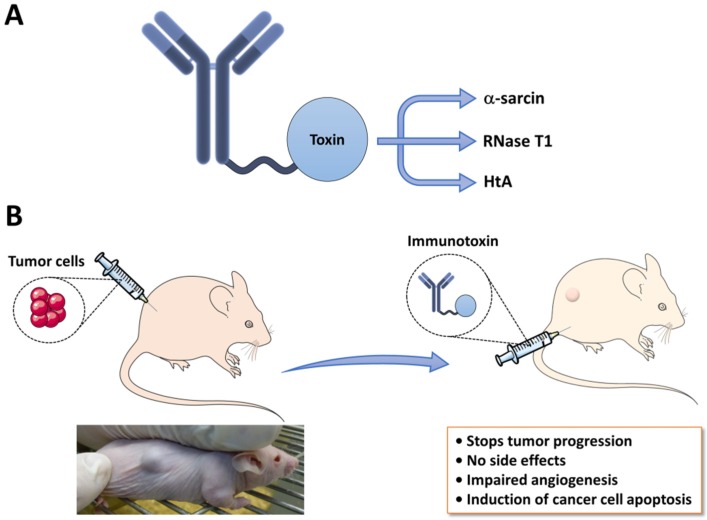
Ribotoxins as immunotherapeutic agents. (**A**) General structure of an immunotoxin, composed of a specific antibody fragment responsible for targeting to a specific cell type linked to a toxin moiety, ribotoxins or non-toxic ribonucleases like RNase T1, which promote cell death; (**B**) IMTXA33αs, an immunotoxin that contains α-sarcin as the toxic domain, has been tested in nude mice.

Even though the role of ribotoxins in nature is not well determined yet, they already have been tested for their use as therapeutic agents. In fact, they were first discovered as antitumor agents, although further studies revealed an unspecific cytotoxicity against non-tumor cells [69]. Later on, the interest for ribotoxins revived, since they could be used as part of immunotoxins in antitumor therapies. Immunotoxins are usually chimeric molecules composed of a specific antibody fragment, responsible for targeting a specific cell surface antigen, linked to a toxin moiety that promotes cell death (Figure 4A) [70,71]. To overcome the great size of the full-length antibody, something that could hinder its penetration through solid tumors, new strategies have been developed where only the antibody variable domains are used. Regarding the toxic moiety, several toxins have been used such as ricin, *Pseudomonas* exotoxin A and diphtheria toxin [72]. Despite being highly effective, in many cases they showed undesirable side effects.

Fungal ribotoxins show features that make them appropriate candidates for the construction of immunotoxins: their small size, high thermostability, poor immunogenicity, resistance to proteases and, most importantly, their high efficiency in inactivating ribosomes. Accordingly, several colon cancer-specific immunotoxins containing ribotoxins (α-sarcin) or other fungal RNases (RNase T1) as the toxic component have been designed and characterized [73,74]. HtA has also been used to create an immunotoxin, using an engineered variant that is unable to cross membranes but still retains the ribonucleolytic activity [13]. This variant seems to be innocuous for cells that do not contain the specifically targeted antigen. Therefore, it might be safer, with reduced unwanted side effects during cancer therapy.

The toxicity of an immunotoxin depends on many molecular aspects, such as the antigen binding affinity, internalization rate, intracellular processing, toxin release and intrinsic toxicity. Accordingly, the intracellular trafficking of ribotoxin-based immunotoxins has been studied, showing how they are internalized via early endosomes, following different pathways depending on the toxin used until they reach the cytosol [13,75]. Finally, a step further in the therapeutic use of ribotoxin-based immunotoxins has been done showing the efficiency of the α-sarcin-based immunotoxin IMTXA33αs, which inhibits tumor growth as well as angiogenesis in nude mice harboring colon cancer xenografts [14] (Figure 4B). This result can be considered the proof of concept that immunotoxins based on ribotoxins may be a unique therapeutic tool against different tumor pathologies. Moreover, an immunotoxin containing a deimmunized variant of α-sarcin showing a complete lack of T cell activation in in vitro assays have been recently described [76]. These results support the design of a new generation of therapeutic deimmunized ribotoxins-based immunotoxins.

Apart from their potential antitumor effect, the therapeutic use of immunotoxins has been already described for other multifactorial disorders like HIV infection [77] or allergic reactions [78,79]. Few studies have been focused on directing toxin action to allergen-reactive cells through the use of the high-affinity IgE receptor (FcεRI) as a target [80,81]. Despite of the significant decrease in IgE levels and mast cell degranulation, immunotoxin effects were accompanied by the onset of adverse side effects, presumably due to the high immunogenicity of plant and bacteria toxin-based immunoconjugates. Accordingly, new strategies for allergy treatment based on either non-specific or specific immunotherapies using immunotoxins arise as innovative and potentially effective approaches [82]. In this regard, the design of allergen-toxin conjugates [83,84,85] and α-sarcin-based chimeric molecules against specific immune cells involved in allergic symptoms (e.g., basophils, neutrophils, mast cells and the newly described innate type 2 lymphoid cells) are already ongoing [86,87,88].

### 4.2. Role in Allergy and Aspergillus Infection

Not only ribotoxins can be used as therapeutic agents to alleviate allergy symptoms, but also *Aspergillus* spp. are human opportunistic pathogens that cause respiratory diseases like asthma, ABPA (allergic bronchopulmonary aspergillosis), aspergilloma and severe infections, especially in immunocompromised people. *A. fumigatus* is the etiological agent in 80% of *Aspergillus*-related diseases. The ribotoxin Aspf1 is one of the main allergens of this fungus [89]. Although Aspf1 is not detected during the initial steps of infection, it seems to play a role in allergic-like pathogenic processes and some regions of the protein have been found to be significantly allergenic [90,91]. Diagnosis and immunotherapy against *A. fumigatus* allergy are frequently based on fungal extracts, but these are highly complex mixtures, with hundreds of different components very difficult to standardize, not to mention the high risk of anaphylactic side effects. Improvement of diagnosis and therapeutics has been focused on the recombinant production of allergens. Since the recombinant Aspf1 still maintains its cytotoxic activity, alternative non-cytotoxic ribotoxin molecules have been searched for, produced and characterized, showing that their N-terminal β-hairpin is involved not only in cytotoxicity, but also in at least one allergenic epitope [91]. These non-cytotoxic and hypoallergenic mutant variants of Aspf1 and α-sarcin might be suitable for their use in immunomodulating therapies against *Aspergillus* hypersensitivity and its diagnosis. Indeed, they have been produced in *Lactococcus lactis*, a GRAS (generally regarded as safe) microorganism that could be used in immunotherapeutic protocols for Aspf1-related diseases as a vaccination vehicle [92]. Furthermore, a mouse model of *A. fumigatus* sensitization has been established, that could be suitable for preclinical testing of recombinant allergens and their derivatives [93].

### 4.3. Ribotoxins as Pest Control Agents

The biological role of ribotoxins as insecticides could be used in the design and development of new biopesticides. The world population increases exponentially and it is estimated to reach 9.5 billion people by 2050. This rapid increase of population requires an equal increase in food resources. Every year, pest diseases are the cause of up to 40% losses in agriculture production around the world. For decades, pest control has relied on the use of chemical insecticides, such as the use of DDT (dichlorodiphenyltrichloroethane) in the 1940s. They were in general highly potent, fast acting, cheap to produce and easy to deliver. Still, the poor species specificity, their toxicity and the emergence of pest resistance forced many countries to ban DDT and other chemical pesticides in the 1970s. Resistance to pesticides has increased over the years due to the use of new chemical products. By 1992 more than 500 species of mites and insects had developed resistance. Moreover, the cost of discovering, developing and registering new synthetic pesticides is so high that a new interest in alternative biological control methods has risen. Biopesticides have become an important component of environmentally friendly pest management. However, its use has not yet reached its potential, constituting only 2%–3% of the insecticidal market [94]. The term biopesticides includes those pest control techniques that use microbial organisms (bacteria, fungi, viruses and nematodes) and/or their secondary metabolites like peptides and proteins. It even comprises genetically modified crops that improve their resistance to insect, fungal, viral or herbicide damage [95,96].

Entomopathogenic fungi have been used effectively as biological control agents to manage crop diseases, some of them being commercialized, like *B. bassiana* in the control of numerous insects and flies, or *M. anisopliae* against the mosquito *Anopheles gambiae* [97,98,99]. Interestingly, and as mentioned above, this fungus produces an HtA-like ribotoxin, anisoplin [6]. *H. thompsonii*, the HtA producer, is also a well-known entomopathogenic fungus used in the biological control of the mite *Varroa destructor*, a honey bee ectoparasite [56,97,100]. This fungus has been shown to be even more effective than *B. bassiana* and *M. anisopliae* in some cases [101]. *H. thompsonii* was first registered in 1981 as a mycoacaricide (Mycar) for the control of the citrus mite [102], but formulation problems, lack of field persistence and poor product stability in storage and transport resulted in the loss of this commercial product late in the 1980s. Attempts to overcome these obstacles have been made, evaluating the use of new formulations that improve the delivery, shelf life and field efficacy [103].

Genetic manipulation has also been used to increase virulence of the entomopathogenic fungi in pest control [104]. The first successful demonstration of an increased virulence was the overexpression in the fungus *M. anisopliae* of a protease capable of degrading the insect cuticle. Here the recombinant expression of ribotoxins could also play an important role.

On the other hand, understanding the mechanism of infection of these fungi as well as the virulence factors involved might help in the design of new formulas for pest control. A number of successful biopesticides are based on using compounds produced by the microbes rather than provoking the infection by the whole organism [94]. In this sense, the characterization of the activity of fungal ribotoxins against insects opens the possibility of using them as bioinsecticides, independently or in formulas where other compounds could be combined with the active agent [11,12]. The potential toxicity of ribotoxins against vertebrates could be overcome by the design of new variants such as the HtA Trp-residues mutants [13,32]. These proteins retain the exquisite ribonucleolytic activity against the ribosome but are unable to enter mammalian cells and therefore are much less cytotoxic.

**Figure 5:**
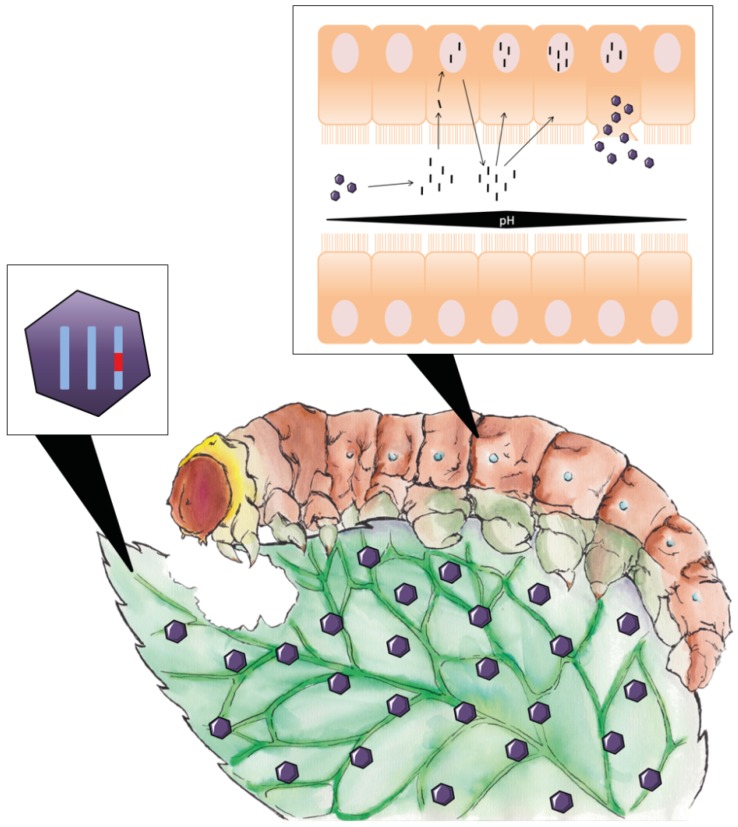
Baculovirus-based biopesticides. Modified baculoviruses containing genes for insect-specific toxins, hormones or other enzymes are sprayed on the plant foliage and then ingested by the caterpillar or adult insect. Polyhedra solubilize in the insect’s midgut due to an increase of pH, virions are released and start replicating within the nuclei of epithelial cells, producing more virions which are released in a budded form (early infection) or occluded in polyhedra (late infection).

Finally, insect pathogenic viruses like baculoviruses represent another strategy for biocontrol (Figure 5). Natural baculoviruses have been used as effective biopesticides, although genetically engineered and recombinantly produced versions seem to be an even better alternative [105,106], as they can kill the host faster without conferring any ecological advantage over the wild-type species. Baculoviruses have the additional advantage of being an easy and stable formula with high specificity for the host. One example related to fungal pathogenesis is the design of an improved baculovirus that expresses a protease from *A. fumigatus* able to induce death in *Spodoptera frugiperda* larvae faster than the wild-type virus [107]. These results encourage the development of similar tools using ribotoxins. Baculovirus engineered to produce fungal ribotoxins, or improved variants of them, would be insect-selective, minimizing side toxic effects against other organisms. The only concern regarding the use of baculoviruses in pest control is the public opinion about the introduction of genetically modified viruses. However, new environmentally friendly versions are already being produced, which consist of viruses that become less active during serial passaging and spontaneously inactive after some time [108].

### 4.4. The Study of Ribosome-Related Diseases. Ribosome Biogenesis as an Additional Target of Ribotoxins

Ribosomes are among the finest molecular machines in the cell. They are essential for life, producing all the proteins required for cell growth. In humans, specific defects especially in their assembly lead to a heterogeneous number of diseases named as ribosomopathies. These diseases have in common a ribosomal dysfunction, but differ significantly in mechanism, clinical symptoms and potential treatments (Table 1). Some disorders, such as Diamond-Blackfan anemia (DBA), have been linked to mutations in ribosomal proteins, whereas others like the Schwachman-Diamond syndrome (SDS) or the Treacher Collins syndrome (TCS) are directly related to defective ribosome biogenesis [15,16,109,110]. Clinical features of these ribosomopathies normally include bone marrow failure, developmental abnormalities and an elevated risk of cancer, although symptoms are diverse even among patients with the same disease [15]. There are many unanswered questions regarding ribosomopathies. Among them, it is quite striking how, being that the ribosome is such a conserved machinery, mutations in ribosomal proteins can lead to such diversity of phenotypes and how sometimes these mutations affect only specific tissues or cell types [16,111]. The elucidation of the basis of these ribosome-related diseases requires a better knowledge of the ribosome, especially its biogenesis, which seems to be altered in a relatively high number of ribosomopathies. In addition, in these studies, *Saccharomyces cerevisiae* has been an important model organism [112,113].

**Figure 6:**
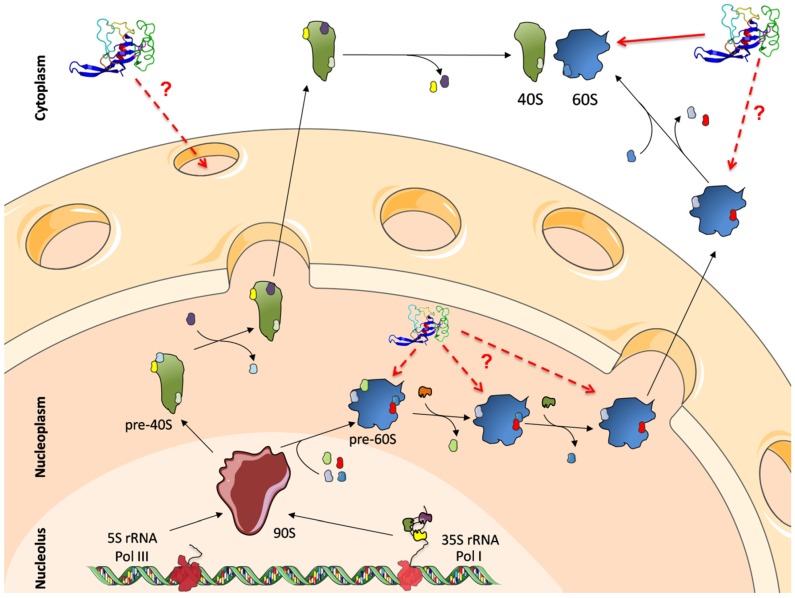
Ribosome biogenesis and pre-rRNA processing pathways in yeast. Schematic representation of ribosome maturation, that starts with RNA Pol I and III transcribing 35S and 5S pre-rRNAs, respectively. These rRNAs associate to ribosomal proteins and other factors building the 90S particle. After cleavage of 35S pre-rRNA, the 90S particle separates into a pre-60S (blue) and a pre-40S subunit (green), which undergo independent maturation. Maturation driven by numerous assembly factors occurs while these particles travel through the nucleoplasm. Once in the cytoplasm final maturation yields translational competent ribosomal particles. Ribotoxins target mature 60S subunits, but pre-60S subunits and the whole ribosome biogenesis pathway might also be affected by their toxicity. Adapted from [144].

Ribosome biogenesis is a complex cellular process where the large and small ribosomal subunits are assembled. In eukaryotes, this process starts in the nucleolus. Then, both subunits undergo maturation through the nucleoplasm and the cytoplasm (Figure 6). More than 300 factors, including proteins and RNAs are involved in the production of a mature ribosome in the yeast *S. cerevisiae*. In humans, this number is even higher, in accordance to the bigger size and complexity of human ribosomes [110,140]. This complex maturation process begins with transcription of 5S and 35S pre-rRNA in the nucleolus, acquiring post-transcriptional modifications and associating with ribosomal proteins as well as other *trans*-acting factors to form the 90S particle. This particle is then cleaved, releasing pre-40S and pre-60S particles that undergo independent maturation pathways. Pre-40S are rapidly exported to the cytoplasm with relatively few compositional changes. Maturation of the pre-60S particle is much more complex and involves several changes of composition in the nucleoplasm. Once in the cytoplasm, major events include the release of factors from the exit tunnel, the assembly of the stalk structure (ribosomal proteins P0, P1 and P2), and the release of the anti-association factor Tif6 (eIF6 in humans), by Sdo1 and the GTPase Efl1 [112,113,141,142,143,144]. Mutations in the human orthologue of Sdo1, SBDS, are responsible for most of the cases of the Shwachman-Diamond syndrome [109,145].

Thanks to powerful techniques like the tandem affinity purification, combined with mass spectrometry, the knowledge of ribosome biogenesis has significantly increased over the past 20 years. However, many questions regarding this complex event are still unanswered. Fungal ribotoxins may shed some light in this sense. From their point of view, it would be interesting to see whether these toxins can access the nucleus, and if they do, whether the SRL of pre-60S particles can be cleaved (Figure 6). From the ribosome biogenesis side, they might be appropriate tools to explore the maturation pathway of pre-60S particles, especially in the cytoplasm, since their target, the SRL, is localized in a region essential during this process. In fact, Efl1 binds to the SRL to release the anti-association factor eIF6 [146]. Moreover, understanding how ribotoxins affect mature and immature ribosomes may give some information about pre-rRNA processing and folding, and the function and localization of some *trans*-acting factors. Eventually, these studies may contribute to a better understanding of pathologies like the already mentioned Shwachman-Diamond syndrome and other ribosomopathies.

## 5. Concluding Remarks

Over the past decades, the structure and function of fungal ribotoxins have been extensively studied. Today, the accumulated knowledge of this family of toxins has allowed their successful use in some downstream applications, like the design of new specific immunotoxins against colon cancer and the establishment of mouse models for the study of aspergillosis. However, the range of potential applications does not end here. The discovery of HtA supports the idea that, in nature, ribotoxins have a major role as defensive toxins with insecticidal activity. Their high efficiency against insect cells and larvae make them good candidates for the design and development of new biopesticides. Furthermore, ribotoxins could be also used in the field of ribosome biogenesis, contributing to a better understanding of ribosomopathies.

## Figures and Tables

**Table 1:** List of gene defects associated to ribosomopathies and their related diseases (Adapted from [114]).

Gene Defect	Impaired Function	Disease	Clinical Features	Treatment	References
RPS19, RPS26, RPL5, RPL11 and other RPs	Different steps of pre-rRNA processing	Diamond Blackfan anemia (DBA)	Anemia, bone marrow failure, growth retardation, congenital abnormalities (craniofacial, thumb), cardiac defects, cancer predisposition.	Corticosteroids, blood transfusions, hematopoietic stem cell transplantation	[115,116,117,118,119,120,121]
RPS14	18S pre-rRNA processing	5q-syndrome	Severe macrocytic anemia, cancer predisposition	Lenalidomide	[122,123]
SBDS	Maturation and export of the 60S ribosomal subunit	Shwachman-Diamond syndrome (SDS)	Exocrine pancreas insufficiency, hematologic defects, skeletal abnormalities, cancer predisposition	Pancreatic enzyme supplementation, hematopoietic stem cell transplantation	[124,125,126]
DKC1	Telomerase deficiency, disease aggravated by box H/ACA snoRNP pseudouridylation defects, involved in pre-rRNA modification.	X-linked dyskeratosis congenita (DC)	Mucocutaneous abnormalities, pulmonary fibrosis, bone marrow failure, cancer predisposition	Oxymetolone, Hematopoietic stem cell transplantation	[127,128,129]
RMRP	Maturation of 5.8S rRNA of 60S ribosomal subunit; degradation of cell-cycle regulated RNAs; mitochondrial DNA replication	Cartilage-hair hypoplasia (CHH)	Short stature, hair hypoplasia, bone deformities, cancer predisposition	Symptomatic	[130,131]
TCOF1	rDNA transcription and methylation of 18S rRNA	Treacher-Collins syndrome (TCS)	Craniofacial abnormalities	Symptomatic	[132,133,134]
EMG1	Maturation of 40S ribosomal subunit	Bowen-Conradi syndrome	Severe growth retardation	None	[135,136,137]
hUTP4/Cirhin	Maturation of the 18S pre-rRNA	North American Indian childhood cirrhosis	Biliary cirrhosis, lethal by adolescence without liver transplant	Liver transplantation	[114,138,139]
